# Coral Fluorescent Proteins as Antioxidants

**DOI:** 10.1371/journal.pone.0007298

**Published:** 2009-10-06

**Authors:** Caroline V. Palmer, Chintan K. Modi, Laura D. Mydlarz

**Affiliations:** 1 School of Biology, Newcastle University, Newcastle upon Tyne, United Kingdom; 2 ARC Centre of Excellence for Coral Reef Studies and School of Marine and Tropical Biology, James Cook University, Townsville, Queensland, Australia; 3 Integrative Biology, University of Texas at Austin, Austin, Texas, United States of America; 4 Department of Biology, University of Texas at Arlington, Arlington, Texas, United States of America; University of North Carolina at Chapel Hill, United States of America

## Abstract

**Background:**

A wide array of fluorescent proteins (FP) is present in anthozoans, although their biochemical characteristics and function in host tissue remain to be determined. Upregulation of FP's frequently occurs in injured or compromised coral tissue, suggesting a potential role of coral FPs in host stress responses.

**Methodology/Principal Findings:**

The presence of FPs was determined and quantified for a subsample of seven healthy Caribbean coral species using spectral emission analysis of tissue extracts. FP concentration was correlated with the *in vivo* antioxidant potential of the tissue extracts by quantifying the hydrogen peroxide (H_2_O_2_) scavenging rates. FPs of the seven species varied in both type and abundance and demonstrated a positive correlation between H_2_O_2_ scavenging rate and FP concentration. To validate this data, the H_2_O_2_ scavenging rates of four pure scleractinian FPs, cyan (CFP), green (GFP), red (RFP) and chromoprotein (CP), and their mutant counterparts (without chromophores), were investigated. *In vitro*, each FP scavenged H_2_O_2_ with the most efficient being CP followed by equivalent activity of CFP and RFP. Scavenging was significantly higher in all mutant counterparts.

**Conclusions/Significance:**

Both naturally occurring and pure coral FPs have significant H_2_O_2_ scavenging activity. The higher scavenging rate of RFP and the CP *in vitro* is consistent with observed increases of these specific FPs in areas of compromised coral tissue. However, the greater scavenging ability of the mutant counterparts suggests additional roles of scleractinian FPs, potentially pertaining to their color. This study documents H_2_O_2_ scavenging of scleractinian FPs, a novel biochemical characteristic, both *in vivo* across multiple species and *in vitro* with purified proteins. These data support a role for FPs in coral stress and immune responses and highlights the multi-functionality of these conspicuous proteins.

## Introduction

Pigments and coloration patterns in nature are both numerous and diverse [1], with an equally diverse set of functions and roles. Pigment functions include visual stimuli; as a warning to predators or to attract con-specifics [1], direct protection from solar radiation and in immune resistance [2]. Invertebrate pigments include small molecules such as carotenoids [3], [4], insoluble polymers such as melanin [5], [6], [7], or proteins such as those involved in bioluminescence and fluorescence [8] . Corals are renowned for their vivid coloration [9], [10], for which fluorescent proteins (FPs) are largely responsible [8]. FPs are abundant and diverse within anthozoans, ranging across four basic color types; cyan (CFP), green (GFP), red (RFP) and a blue/purple non-fluorescent chromoprotein [11], [12], [13], however their function within the holobiont remains undetermined and controversial [14], [15]. Analogous to other natural pigments, the differential variation of FPs [9], [16], [17], [18], including temporally [19], [20], [21] and spatially [15], [22], [23] within a colony, suggests multiple specific roles [18], [24], [25].

The most prominant hypotheses of FP function within corals are related to the maintenance of the obligate symbiosis with dinoflagellates, commonly known as zooxanthellae. The ability of FPs to convert shorter wavelengths of light into longer wavelengths has led to suggested photoprotective [26], [27], [28], [29] and light enhancing roles [9], [24]. Although the spectral properties of some FPs potentially support these hypotheses [30], [31], [32], spectral emission of the red FP does not [33]. This combined with the histological location of red FP in the epidermis of compromised tissue [23], as well in tissues with equivalent light environments to tissues without red FP [23] and their presence within azooxanthellate organisms [34], implies additional roles. Other FP functions that have been proposed include use as visual triggers for other organisms [35], [36] and as oxygen radical quenchers [12], [37].

Reactive oxygen species (ROS) exposure is continuous for aerobic organisms whether as part of normal cell function, from exogenous sources [38] or during stress responses [39], [40], [41], [42], [43]. Oxygen radicals are readily produced by a number of pathways and mechanisms [44] including by algal symbionts [45], [46], therefore regulation of tissue redox state is an important mechanism for the zooxanthellate scleractinian corals.The photosynthetic zooxanthellae generate high quantities of dissolved oxygen under normal conditions [42], but during thermal and light stress events ROS levels are elevated [42], [45], inducing oxidative stress [38] in both the symbiont and the coral host [42], [47]. In addition, the coral response to thermal stress [43], injury [44], [48] and infection [23], [49], [50] also contributes to elevated ROS in host tissues. Of the ROS, hydrogen peroxide (H_2_O_2_) is particularly attributed to the induction of oxidative stress [51], as it is the most stable of the oxygen species and easily diffuses across biological membranes [52], [53].

To mitigate and regulate ROS cytotoxicity anthozoans possess a defensive suite of endogenous antioxidant enzymes [39], [54], [55] such as superoxide dismutase [56], [57] which catalyzes the conversion of superoxide anion to water and H_2_O_2_ [39], catalase [51], [58], [59] which catalyses the decomposition of H_2_O_2_ to water and oxygen [60] and peroxidases which are oxidant proteins that consume H_2_O_2_ [50]. In addition, invertebrates including octocorals, assimilate exogenous antioxidants such as carotenoid pigments into their tissue and skeleton from food sources or symbionts [61], [62]. These conserved antioxidant pathways can be overwhelmed during extreme temperature stress [43], inducing the expulsion of symbionts (bleaching) [45] and during pathogen infections [50] both of which can cause localised tissue mortality. This implies that these well-characterised antioxidants have a physiological limitation, thus requiring supplemental scavenging activity from local proteins.

**Figure 1:**
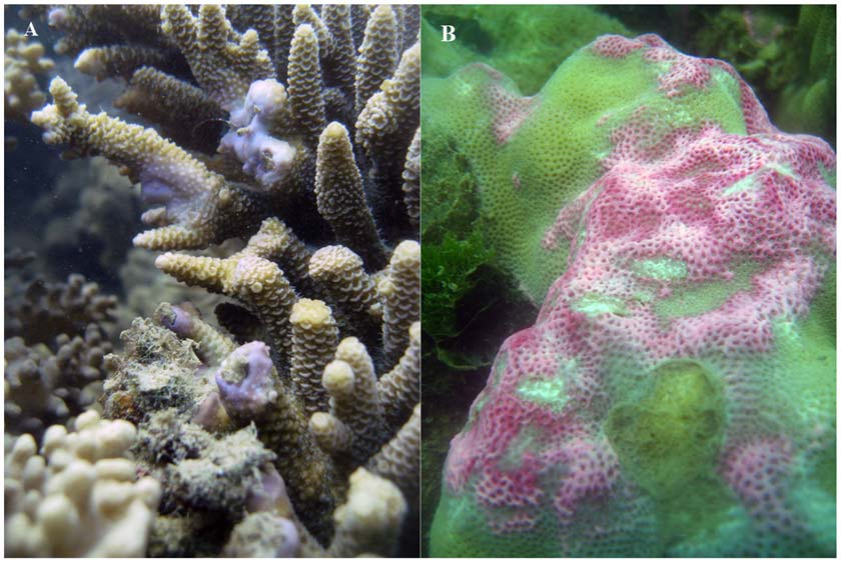
Pigmentation responses of a) *A. millepora* with blue pigmentation in response to breakage and b) *Porites* massive sp. with pink pigmentation in response to fish bites.

Coral tissue that has been compromised by injury [49], [63] and infection [23] frequently develops localised non-normal pigmentation, for example of blue/purple coloration in *Acropora* sp. (fig. 1a) and pink/red in *Porites* sp. (fig. 1b) [23], [49] of which an RFP has recently been found to be responsible [23]. In addition, non-normally pigmented areas of compromised tissue demonstrate increased activity of the melanin-synthesis pathway [49] and thus increased abundance of oxygen radicals [7]. A potential role of FPs as radical quenchers in anthozoan host stress responses may explain the temporal and spatial localised variation in FP type and concentration [9], [23].

Despite their prevalence among corals the basic biochemical characteristics are largely unknown and potential function(s) of FPs remain unresolved [14], [36]. In this study we document the diversity and quantify the abundance of FPs within multiple Caribbean coral species. The investigation of these primary reef-framework builders is both timely and vital given the rapid decline of Caribbean corals due to disease and bleaching [64]. In this study, we test the hypothesis that FPs have antioxidant capabilities by examining the H_2_O_2_ scavenging potential of coral tissue extracts and the relationship to FP concentration, *in vivo*. We also examine the H_2_O_2_ scavenging abilities of four pure scleractinian FPs and their colorless mutants *in vitro*.

## Methods

### Samples and tissue extraction protocol

Three genetically distinct fragments of seven Caribbean hard coral species, *Montastraea annularis, Montastraea faveolata, Montastraea cavernosa, Diploria strigosa, Porites astreoides, Dichocoenia stokseii* and *Sidastrea siderea* were collected from the National Oceanic and Atmospheric Administration/Florida Keys National Marine Sanctuary (NOAA/FKNMS) Coral Nursery in Key West FL in May of 2007. The fragments were obtained and transferred to Mote Tropical Marine Laboratory under the specifications of research permit number FKNMS-2007-050, snap frozen in liquid nitrogen and shipped on dry ice to the University of Texas at Arlington where they were stored at −80°C.

Additionally, during April 2009 three colonies of *Acropora millepora* with distinct blue pigmentation as a result of physical breakage were identified (fig. 1a) together with three *Porites* massive species with intense pink pigmentation (fig. 1b) in Pioneer Bay, Orpheus Island on the Great Barrier Reef (GBR). A subsample of the pigmented tissue was removed from each colony and an equivalent area of healthy tissue. All samples were snap frozen in liquid nitrogen and processed for enzyme assays as below. Samples were stored at −30°C.

Tissue was removed from all of the frozen samples with an airbrush (Paansche) and extraction buffer (50 mM phosphate buffer, pH 7.8 with 0.05 mM dithiothreitol) over ice. The tissue slurry was homogenised with a medium sawtooth (Fisher Scientific, Power Gen 125) for 20 s and left on ice for 5 minutes to extract the proteins. Samples were then vortexed with a spatula of glass beads for 20 s and left on ice for another 5 minutes. Tissue slurries were centrifuged at 4°C at 10,000 RPM for 5 minutes to remove the supernatant from the cellular debris and stored at −80°C until use.

### Coral spectral emission

Aliquots of 30 µl of each sample were added in triplicate to a black/clear 384 well microtitre plate with parallel aliquots of extraction buffer to control for independent effects. Each well was excited at 280 nm using a spectrophotometer (SpectraMax M2, Molecular Devices), and the emission spectra measured in 5 nm increments from 400 nm to 650 nm for the Caribbean coral samples. For the GBR *Porites* massive samples each well was excited at 540 nm and the emission between 570 nm and 590 nm recorded. Additionally, for the *A. millepora* GBR samples, the endpoint absorbance at 588 nm was recorded for each well. Relative fluorescence, and absorbance, of each sample was standardised to the sample's total protein concentration as determined by the Quick Start Bradford protein assay (Bio-Rad). The validity of this standardisation for fluorescent proteins was tested by measuring the fluorescence of a serial dilution of protein-quantified tissue extract (r^2^ = 0.956). The background scatter for each fluorescent emission spectra was removed by creating individual baseline curves from three points of the spectra, 400 nm, a baseline mid point and at 600 nm points, and solving for the exponential (y = e^mx^). This was then subtracted from each RFU value. The total fluorescence per mg protein for each sample was calculated by summing the standardised RFUs between 465 nm and 600 nm. Total fluorescence was compared between coral species using a one-way ANOVA, as assumptions of normality and homogeneity of variance were met or the non-parametric Kruskal-Wallis test where assumptions were not met.

### Coral H_2_O_2_ scavenging

Using the same tissue extract as described above, 20 µl aliquots were added in triplicate to wells of a 96 well UV transparent microtitre plate (Costar). To each well 30 µl of phosphate buffer (pH 7.0, 0.05 M) and 50 µl of 50 mM H_2_O_2_ were added and the absorbance at 240 nm read immediately and every 31 seconds for 8 minutes. Sample blanks were used to control for independent sample effects. The mean mM H_2_O_2_ scavenged was calculated by subtracting the final absorbance from the initial and related back to mM H_2_O_2_ using a standard curve run (serial dilution from 50 mM to 3.125 mM) on the same plate. Scavenging activity was normalized to mg protein for each sample. Mean scavenging rates of each species were compared using a two-way ANOVA and Tukey post-hoc tests for Caribbean coral samples and student t-tests for GBR samples, the spectral emission data was log transformed to satisfy parametric analysis constraints. The correlation between the rate of H_2_O_2_ scavenging and the relative proportion of the summed standardised fluorescence for each color (cyan = 465 to 500 nm, green = 505 to 550 nm and red = 555 to 600 nm) was analysed using regression analysis.

### Expression of fluorescent and mutant proteins in E. coli

The bacterial expression constructs for *A. millepora* FPs were designed previously [13] according to the protocol outlined in Kelmanson and Matz (2003). Briefly, the constructs were based on pGEM-T vector (Promega, WI, USA), into which a PCR-amplified fragment bearing a full Open Reading Frame (ORF) of a fluorescent protein was inserted, in an orientation corresponding to the transcription from the vector's *lac* promoter. The primers used for amplifying the ORF additionally encoded essential translation initiation signals and 6xHis tag at the C-terminus of the protein for affinity purification. Plasmids were transformed in to Z-Competent E. *coli* cells (Zymo Research, CA, USA) and plated on LB/Agar plates with 50 µg/ml Ampicillin and 1 µM isopropyl-β-D-1-thiogalactopyranoside (IPTG). After confirming fluorescent and non-fluorescent colonies under a Leica MZ FL III stereomicroscope using Chroma filter (set #11003 BL/VIO) for each construct, approximately 30 transformed colonies were picked into 0.5 ml microcentifuge tubes containing 50 µl of Super Optimal Broth (SOC) media with Ampicillin. Cell suspension for each of the constructs were streaked with toothpicks onto four large Luria-Bertani (LB) media/Agar plates with Ampicillin and IPTG. Plates were inverted and incubated at room temperature for 3–4 days, to achieve for maximal fluorescence or color development. The cells expressing the non-fluorescent mutants grew slower; therefore, these plates were incubated for 5–6 days. For control, a dummy protein expression and purification was performed using Z competent cells transformed with only pGEM-T vector with no insert.

### Mutagenesis of fluorescent proteins from *Acropora millepora*

Non-fluorescent mutants of three FPs previously cloned from *Acropora millepora* [13] were made, including the native fluorescent cyan (amilCFP), green (amilGFP), red (amilRFP) proteins. In all cases, the key chromophore-forming tyrosine residue (corresponding to Y66 in GFP from *Aequorea victoria*) was replaced by alanine. This substitution completely abolished the chromophore synthesis without disrupting the structure of the protein. The mutagenesis was carried out with QuikChange II Site-Directed Mutagenesis kit (Stratagene, CA, USA), following the provided protocol, and using associated software for designing the oligonucleotides. Mutant constructs were transformed in to *E. coli*, Top10 chemical competent cells (Invitrogen, CA, USA), and several non-fluorescent clones were Sanger-sequenced to confirm the success of mutagenesis.

### Protein isolation and purification

Cells were harvested from each plate using 10 ml of 1 x phosphate buffered saline (PBS). Cell suspensions were frozen at −80°C and then thawed quickly at 42°C to lyse the cells, followed by sonication using Misonix Sonicator 3000 with microtip (alternating 30 sec. pulses with intensity 6.5 and 30 sec. rest periods, while keeping the tube in an ice bath). The cell lysate were centrifuged at 3900 RPM for 30 min in Eppendorf centrifuge 5810R at 8°C. Cleared supernatants were transferred into new 50 ml conical tubes.

The FPs and their mutants were purified from the supernatants using the two-step protocol consisting of three-phase extraction [65] followed by affinity chromatography using Qia-Expressionist protocol and Ni-NTA agarose (Qiagen, CA, USA). Protein was eluted with 0.5 M imidazole-Na in 1 x PBS, pH 7.0. This buffer was replaced by 1 x PBS via three cycles of concentration-redilution in Ultra-15 centrifuge concentrators with Ultracel-10K filters (Amicon, MA, USA); after which the proteins were concentrated within the final volume of 250–500 µl. Purified and concentrated preparations were stored at 16°C. The purity of the resulting preparations was evaluated by SDS-PAGE on 4–15% Tris-HCl gel (Bio-Rad).

### Pure FP H_2_O_2_ scavenging in vitro

Pure fluorescent proteins were diluted to 2.5 mg ml^−1^ protein in phosphate buffer (pH 7.8, 50 mM) and 10 µl aliquots of each were added in triplicate to wells of a 96-well UV transparent microtitre plate. To each well 40 µl of phosphate buffer (pH 7.0, 0.05 M) and 50 µl of 100 mM hydrogen peroxide were added and the absorbance at 240 nm read immediately and every 31seconds for 1.5 minutes. Sample blanks were used to control for independent sample effects and samples were standardised to a H_2_O_2_ standard curve using a serial dilution from 50 mM to 3.125 mM. The rate of H_2_O_2_ scavenging was compared between samples using a two-way ANOVA with Tukey HSD, as assumptions of normality were met.

## Results

### Caribbean coral spectral emission

**Figure 2:**
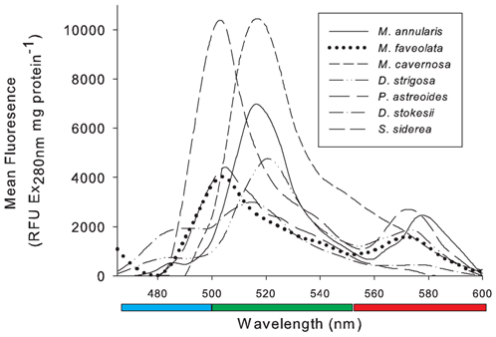
The mean fluorescent emission spectra per mg protein for seven Caribbean coral species (ex. 280 nm).

The spectral emission differed for all seven Caribbean coral species (fig. 2) indicating inter-specific variation in the presence and concentrations of each FP. CFP (465 nm to 500 nm) is present only in *M. annularis*, *D. strigosa* and *D. stokseii* and as small peaks of less than 2000 RFU mg protein^−1^. All species had fluorescent peaks within the green spectra, indicative of the presence of GFP-like proteins, at either approximately 505 nm or 515 nm. Of these, *S. siderea* and *M. cavernosa* had the highest amounts of GFP at approximately 10000 RFU per mg protein and *D. stokseii* the lowest at approximately 3000 RFU per mg protein. Four of the seven corals, *S. siderea, M. annularis, D. strigosa* and *M. faveolata*, had broad peaks of relatively low magnitude (<3000 RFU mg protein^−1^) between 575 and 590 nm, and slight peaks were detectable for *P. astreoides* and *D. stokseii*, indicating the presence of RFP. Additionally, *M. cavernosa* had a broad shoulder extending from the GFP peak at approximately 540 nm through the red spectrum. Despite the variation in emission spectra, the mean total fluorescence (RFU from 465 nm to 600 nm, table 1) did not significantly different between species (F _(6, 19)_ = 0.468, P = 0.82). However the three species with the highest fluorescent peaks, *M. cavernosa*, *S. siderea* and *M. annularis* had the highest mean summed fluorescence, although with high variation. *M. faveolata, P. astreoides* and *D. stokseii* had the lowest mean summed fluorescence. Concomitantly, mean summed RFU per mg protein for CFP, GFP and RFP did not differ between species (F _(6, 19)_ = 0.489, P = 0.806l; K-W χ^2^ = 1.84, P = 0.93 and K-W χ^2^ = 9.55, P = 0.145 respectively, table 1).

**Table 1:** The mean summed total fluorescence (RFU mg protein^−1^), CFP, GFP and RFP for each species.

Species		Total	Cyan	Green	Red
***M. annularis***	***Mean***	53900	3548.3	37780.7	11910.6
	*Std. Err.*	49567.2	3548.3	34396.0	10973.5
***M. faveolata***	***Mean***	38405.4	8304.1	20846.4	8803.8
	*Std. Err.*	21757.0	7968.2	13664.3	1240.9
***M. cavernosa***	***Mean***	82575.6	3575.9	64956.8	13466.2
	*Std. Err.*	60383.6	3321.6	46514.2	9977.3
***D. strigosa***	***Mean***	44328.9	4779	28692.8	10646.5
	*Std. Err.*	17188.9	4116.8	13801.2	346.6
***P. astreoides***	***Mean***	33736.4	8042.9	23795.4	1898.0
	*Std. Err.*	9965.5	3798.1	6903.1	800.0
***D. stokesii***	***Mean***	33482.4	11052.2	19174.6	3166.4
	*Std. Err.*	15771.6	9594.3	6117.0	366.5
***S. siderea***	***Mean***	77430.8	21224.4	41853.5	13886.4
	*Std. Err.*	42543.9	12250.9	25364.2	5428.7

### Caribbean coral H_2_O_2_ scavenging

**Figure 3:**
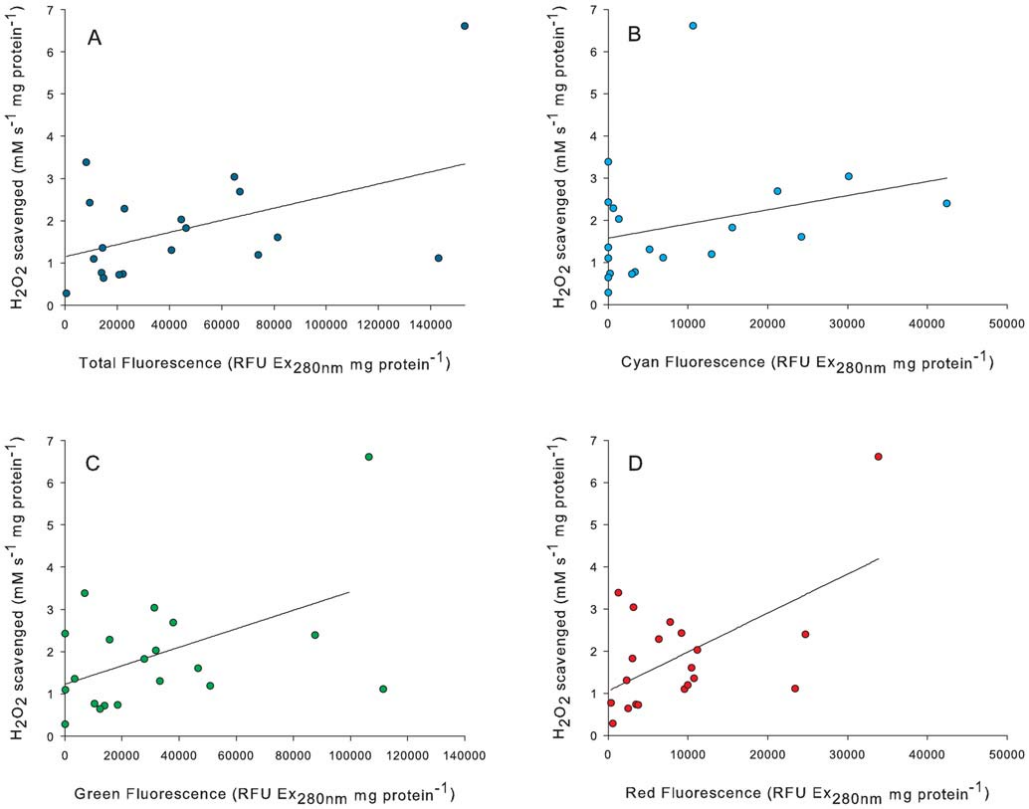
The correlation between the fluorescence and the rate of H_2_O_2_ scavenged for a) total RFU from 465 nm to 600 nm (R^2^ = 0.25, F = 6.05, P = 0.024), b) CFP from 465 nm to 500 nm (R^2^ = 0.08, F = 1.6295, P = 0.218), c) GFP from 505 nm to 550 nm (R^2^ = 0.22, F = 4.95, P = 0.04) and d) RFP from 555 nm to 600 nm (R^2^ = 0.34, F = 9.37, P<0.01).

The mean rate of H_2_O_2_ scavenged per mg protein was not significantly different between the seven Caribbean coral species (F _(6, 19)_ = 1.035, P = 0.45, table 2). *M. annularis* and *S. siderea* had the highest scavenging rate at 3.4 and 2.5 mM s^−1^ mg protein^−1^, respectively. *M. cavernosa* had the lowest with 0.9 mM s^−1^ mg protein^−1^. There was a significant positive relationship (R^2^ = 0.25, F _(1, 19)_ = 6.05, P = 0.02) between the H_2_O_2_ scavenging rate and the total fluorescence (465 nm to 600 nm, fig. 3a). For the constituent fluorescent proteins, H_2_O_2_ scavenging rate demonstrated no significant relationship with CFP (465 nm to 500 nm) (R^2^ = 0.08, F _(1, 19)_ = 1.63, P = 0.22, fig. 3b). H_2_O_2_ scavenging rate and GFP (505 nm to 550 nm) had the second strongest and significant correlation (fig. 3c) with R^2^ = 0.22 (F _(1, 19)_ = 4.95, P = 0.04), and RFP (555 nm to 600 nm, fig. 3d) the strongest (R^2^ = 0.34 (F _(1, 19)_ = 9.37, P<0.01).

**Table 2:** The mean H_2_O_2_ scavenging rate (mM s^−1^ mg protein^−1^) and standard errors for each coral species.

Species	Mean	Std. Err.
*M. annularis*	3.4	1.8
*M. faveolata*	1.7	0.3
*M. cavernosa*	0.9	0.2
*D. strigosa*	1.5	0.3
*P. astreoides*	1.3	0.3
*D. stokesii*	1.5	0.8
*S. siderea*	2.5	0.1

### GBR coral fluorescence and H_2_O_2_ scavenging

**Figure 4:**
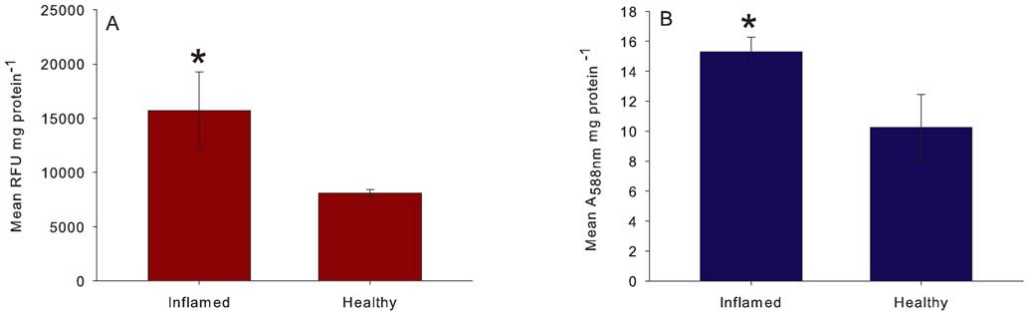
The mean relative FP concentration in inflamed and healthy tissue for a) RFP (RFU±SE) in *Porites* sp. (T _(2)_ = 13.0067, P = 0.05) and b) CP (absorbance at 588 nm±SE) in *A. millepora* (T _(2)_ = 4.0497, P = 0.05).

**Figure 5:**
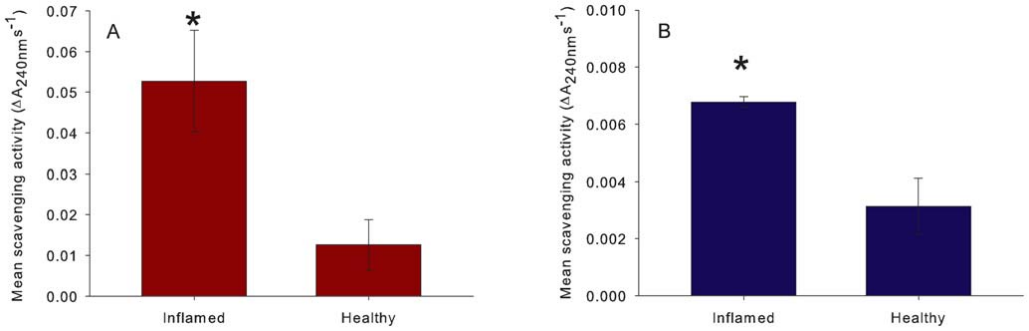
The mean H_2_O_2_ scavenging activity (±SE) of healthy and inflamed tissue of a) *Porites* sp. (T _(4)_ = 2.8675 P = 0.05) and b) *A. millepora* (T _(4)_ = 3.6235, P = 0.02).

The mean relative fluorescence of non-normally pigmented, inflamed *Porites* massive tissue (fig. 4a) was significantly higher than that of healthy tissue (T _(2)_ = 13.0067, P = 0.05). Additionally, the mean absorbance at 588 nm was significantly higher for inflamed tissue as compared to healthy tissue of *A. millepora* (T _(2)_ = 4.0497, P = 0.05, fig. 4b). For H_2_O_2_ scavenging, there was a significantly higher activity for the inflamed, pigmented tissue of both species (fig. 5a and b) as compared to their respective healthy tissues (t _(4)_ = 2.8675, P = 0.05 for *Porites* and t _(4)_ = 3.6235, P = 0.02 for *A. millepora*). Additionally, the *Porites* inflamed tissue scavenging activity was 10-fold that of the inflamed tissue of *A. millepora*.

### Pure FP H_2_O_2_ scavenging in vitro

**Figure 6:**
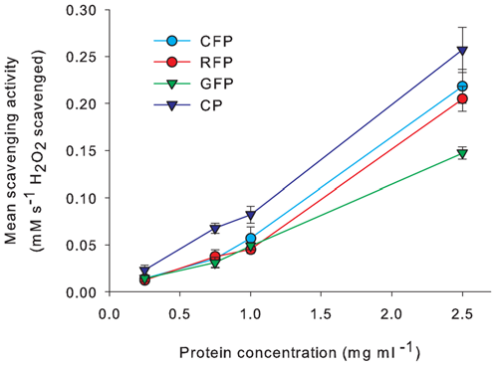
The mean mM H_2_O_2_ scavenged (±SE) for each pure fluorescent protein at a series of concentrations (F _(3)_ = 10.81, P<0.001).

**Figure 7:**
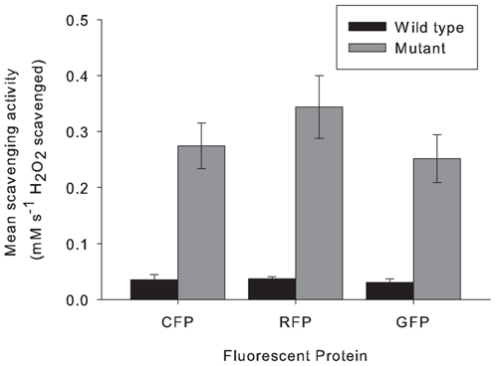
The mean mM of H_2_O_2_ scavenged (±SE) per mg of protein for each FP and the corresponding mutant (Wild type compared to mutant F _(5)_ = 34.4, P<0.001).

The pure (wild-type) FPs all demonstrated significant dose-dependant scavenging activity (F _(3)_ = 233.42, P<0.001; fig. 6), which differed significantly between FP type (F _(3)_ = 10.81, P<0.001) with CP scavenging the highest amount of H_2_O_2_ at each concentration, followed by CFP and RFP. The GFP has the lowest scavenging activity at the highest concentration. Mutants of GFP, CFP and RFP scavenged H_2_O_2_ (fig. 7) up to five-fold of their wild type counterparts (F _(5)_ = 34.4, P<0.001), with no significant difference between FP (P = 0.17). CP mutants were not examined in this study. Procedural controls, i.e blank buffer as well as mock protein expression and purification from Z competent cells transformed with empty pGEM-T vector did not demonstrate any scavenging activity.

## Discussion

This study provides preliminary evidence that coral FPs scavenge hydrogen peroxide (H_2_O_2_) both *in vivo* and *in vitro*, and thus describes a novel biochemical characteristic for these conspicuous proteins. Antioxidants are vital in the avoidance of lipid and DNA peroxidation and other damaging cellular effects [4]. In extreme conditions, such as prolonged temperature stress or pathogen and parasite invasion, the well-characterized and conserved antioxidant pathways, including catalase and superoxide dismutase, may be overwhelmed [38], [66]. This study identifies an additional role of FPs as supplemental antioxidants which may work to prevent oxidative stress in coral tissue and further supports the hypothesis that FPs serve multiple functions within anthozoans [18].

### Coral spectral emission

Caribbean reefs are in rapid and significant decline [67], [68], [69], [70]. Despite the documented increase in emergent diseases [71] and their prevalence, [72] the biological criteria which underlie inter-specific disease susceptibility are yet uncharacterised. Therefore the elucidation of additional immune pathways and resistance mechanisms will undoubtedly lead to a more comprehensive understanding of coral disease resistance [49], [72], [73]. The improvement on spectral emission standardisation and quantification methods in this study [74] enabled the direct comparison of FP type and concentration in seven Caribbean coral species, highlighting inter-specific differences. Consistent across all seven species however, is the presence of GFP, supporting previous reports of GFP as a common coral FP [12], [36]. Despite this, the differing emission spectra for each species demonstrates the diversity and variation of FPs between scleractinian corals, reflecting the variation in host pigmentation directly observed on the reef [8], [9], [16], [17], [18].

Statistically, the levels of total FP mg protein^−1^ did not differ among the Caribbean coral species, potentially attributable to high within species variability, low sample sizes, and low resolution of the protocol. Combining the technique described here, with gene expression tools may overcome these limitations. Further, it is well documented that coral FP expression is dynamic [9], [15], [18], [22] therefore it may not be expected that healthy corals differ in total FP concentrations as clearly as comprised corals do. It is also likely that corals exhibit temporal patterns in FP concentrations and our sampling design of one time point per coral would mask differences that could be temporally detectable. Therefore, observing corals over time would be more indicative of total FP fluctuations between and within coral species.

The species used in this study represent corals with differing life history strategies and disease susceptibilities. Our study included members of the genus *Montastraea* which represent the main framework builders on many Caribbean reefs [75]. However, populations are in decline [70] with *M. annularis* and *M. faveolata* currently listed as “endangered” on the IUCN Red List [76] partly due to their susceptibility to many of the characterised Caribbean diseases [72]. In contrast, populations of *M. cavernosa* are not declining as rapidly and have been listed as a species of “least concern”[77]. *M. cavernosa* has a remarkably higher mean fluorescence than both *M. annularis* and *M. faveolata*, as illustrated in figure 2, although all three *Montastraea* species show a large degree of variability between different genotypes. Members of the *Montastraea* genus are known for their range of colormorphs, especially *M. cavernosa* [18] which may contribute to the extreme intra-specific variability we observed in total FP RFU/mg protein. Even with the propensity for *M. cavernosa* to exhibit different colormorphs, all colonies possess all FP genes, demonstrating that the differential expression is linked with environmental plasticity [18]. Interestingly, *M. faveolata* has much lower overall concentration of FPs, it remains to be seen what relationship this has to its high susceptibility to bleaching and disease [78].

Green and brown color morphs of *P. astreoides* have been previously documented [79], although, unlike the *Montastraea* species, this did not affect the variability in total FP. *P. asteroides* is a very resistant coral, being tolerant to both disease and bleaching [80], [81]. The GFP emission peak for *P. astreoides* in the current study is consistent with other *Porites* species [30]. Also similar to other *Porites* species, *P. asteroides* did not show a strong RFP signal. RFP has only been documented in compromised tissue of other *Porites* species [13], [23] and not in apparent healthy tissue. This reinforces the concept of plasticity and suggests differential utilisation of FPs during stress events.

Spectral emission data on *D. stokseii* and *S. siderea* is documented for the first time in this study. *D. stokesii* has the lowest FP concentration of all seven species, with a low peak of CFP, a slightly higher peak of GFP and a slight peak of RFP. *S. siderea* has emission spectra of similar magnitude to that of *M. cavernosa*. *S. siderea* however has a slightly CFP shifted GFP peak at 505 nm as opposed to the 510 nm peak of *M. cavernosa*, and also a definite peak, but of lower concentration, of RFP. It is not yet clear what the high levels of GFP may be conferring these corals.

### FP H_2_O_2_ scavenging

All Caribbean coral species used in this study showed demonstrable H_2_O_2_ scavenging activity, although inter-specific differences were not statistically significant. However, there was a positive correlation between the total FP and the rate of H_2_O_2_ scavenging by coral tissue extracts. More specifically, RFP and GFP account for the highest amount of H_2_O_2_ scavenging as compared to CFP which did not show a significant relationship and does not conclusively account for any *in vivo* H_2_O_2_ scavenging. Even though our scavenging assays could not distinguish between catalase and FP scavenging in the mixed coral extracts, the significant positive relationship between FP concentration and scavenging activity across a range of corals species provides preliminary evidence for this novel role of anthozoan FPs. H_2_O_2_ scavenging activity was further validated *in vitro* using purified *A. millepora* FPs and all four FPs exhibited dose-dependent H_2_O_2_ scavenging activity, with significant differences among the FPs. CP had the highest activity, followed by CFP and RFP and GFP had the lowest activity. Therefore a role of FP's may be to supplement catalase, the main H_2_O_2_ scavenging protein [59], which can become limited during oxidative stress [38], however further investigation into the molecular mechanisms of this biochemical property is required.

The differing scavenging activity of the different FPs observed in both experiments can be partially explained by the differential allocation of FPs within coral tissue [15], [23]. GFP is found abundantly in the studied species as well as other coral species [82] and consistently throughout the coral tissue [12], [36]. Therefore it is not surprising that GFP accounted for a significant amount of *in vivo* H_2_O_2_ scavenging in the coral species tested. However, since pure GFP was the least efficient H_2_O_2_ scavenger in our *in vitro* assay, it may be that the *in vivo* GFP-scavenging correlation is driven by the high within-tissue concentrations. Conversely, maybe corals need to store higher levels of GFP in their tissues as a result of its less potent scavenging activity.

CFP did not have any *in vivo* scavenging activity, although this result may be due to the relatively low presence of CFP within the seven coral species used in this study. This result was not entirely unexpected since CFPs are limited in their prevalence [82] and primarily located within a relatively small area of tissue on tentacle tips [15]. Since pure CFP does actually have high scavenging activity, the role of CFP as an antioxidant in corals may be spatially and temporally regulated.

RFP was the most efficient *in vivo* scavenger and purified RFP had potent *in vitro* activity as well. RFP is notable since it is identified and upregulated in areas of infected or compromised coral tissue, leaving conspicuous red-pink lesions [23] as confirmed in the present study. Additionally, pure CP was a superior H_2_O_2_ scavenger compared with its fluorescent counterparts and, like RFP, CP causes hyper-pigmentation in compromised tissue of *A. millepora* [49] as confirmed in the current study. CP is also predominantly limited to extremities of colonies, such as branch tips and basal boundaries. Furthermore, compromised tissue with higher FP concentrations, of *A. millepora* and *Porites* massive sp. scavenges H_2_O_2_ more efficiently than the equivalent healthy tissue. This correlation supports the conclusion that FPs have the ability to scavenge H_2_O_2_ *in vivo* and also eludes to the biological significance of FPs as part of innate immunity. These observations demonstrate that FPs with high H_2_O_2_ scavenging efficiency are preferentially upregulated in tissue that is compromised or in frequent contact with foreign organisms [18].

Pigmentation responses are common within the anthozoa, documented in both scleractinian corals [23], [49] and the gorgonian sea fan [50] which become pigmented in areas of injury and infection due to increased FPs [23] and carotenoids [62] respectively. Additionally, the observation that during temperature stress and bleaching, corals have increased fluorescence in their tissues [83] supports roles consistent with photoinduced FP activation [84] and antioxidant potential. FPs are heat-resistant [85] which is a potentially beneficial property during temperature-related oxidative stress, in order to support enzymatic antioxidants which may become overwhelmed or limited [38], [59]. Concomitantly, increased levels of SOD activity have been observed in temperature stressed coral [45], [66] in addition to SOD-like activity documented from a jellyfish GFP [37]. This supports the requirement for increased H_2_O_2_ scavenging in stressed corals as H_2_O_2_ is a product of SOD activity [42]. Therefore, spectrally monitoring the dynamics of FPs potentially provides a valuable and comparatively inexpensive tool for elucidating the relative health status and oxidative state of corals.

Despite the differential scavenging efficiency of the four wild-type FPs and the role of pigments as antioxidants across the metazoa [3], [4], [86], H_2_O_2_ scavenging rates are significantly higher for the non-fluorescent mutant counterparts. While this was unexpected, it highlights the lack of importance of the fluorophore, and therefore the color, of these proteins to their antioxidant activity. This therefore suggests alternative, more dominant roles of FPs than purely as antioxidants, which has enabled the evolution of their diverse color range [17]. This therefore also supports the proposed role of coral FPs as visual triggers for other organisms, such as predatory fish [35], [36]. Overall these findings support the suggestion that FPs serve multiple specific roles and functions that differ between the types of FPs [18], [24], [25].

This study documents H_2_O_2_ scavenging by scleractinian FPs for the first time and therefore proposes an additional role of anthozoan FPs as antioxidants. The diversity, temporal and spatial variation in coral FP distribution and abundance, in conjunction with differential antioxidant potentials, suggests that FP roles may differ between coral species or with changing environmental conditions. Proposed anthozoan FP functions are numerous, potentially dynamic and not mutually exclusive. Further elucidation of these functions will be gained through time series investigations into FP responses to both biotic and abiotic stressors, in addition to molecular modelling to determine the mechanisms of H_2_O_2_ breakdown.
